# Freely designable optical frequency conversion in Raman-resonant four-wave-mixing process

**DOI:** 10.1038/srep08874

**Published:** 2015-03-09

**Authors:** Jian Zheng, Masayuki Katsuragawa

**Affiliations:** 1 Department of Engineering Science, University of Electro-Communications; 2 JST ERATO-IOS 1-5-1, Chofugaoka, Chofu, Tokyo 182-8585, Japan

## Abstract

Nonlinear optical processes are governed by the relative-phase relationships among the relevant electromagnetic fields in these processes. In this Report, we describe the physics of arbitrary manipulation of Raman-resonant four-wave-mixing process by artificial control of relative phases. As a typical example, we show freely designable optical-frequency conversions to extreme spectral regions, mid-infrared and vacuum-ultraviolet, with near-unity quantum efficiencies. Furthermore, we show that such optical-frequency conversions can be realized by using a surprisingly simple technology where transparent plates are placed in a nonlinear optical medium and their positions and thicknesses are adjusted precisely. In a numerical simulation assuming practically applicable parameters in detail, we demonstrate a single-frequency tunable laser that covers the whole vacuum-ultraviolet spectral range of 120 to 200 nm.

Nonlinear optical processes are dominated by the relative-phase relationships among the relevant electromagnetic fields in the processes. The representative example could be phase-matching^1^. Since the birth of nonlinear optics in 1961^1^,^2^, huge efforts have been devoted to studying how we can achieve phase-matching to realize efficient nonlinear optical phenomena. Various technologies have been developed with great success^3^,^4^,^5^, including those that use crystal birefringence^6^,^7^, the angle distributions of the relevant laser fields^8^, the concept of quasi-phase-matching^1^,^9^,^10^, or the control of optical properties by embedding metamaterial structures^11^. However, the control of relative-phase relationship is not necessarily just for achieving phase-matching. In general, there are various possibilities. Although it has barely been discussed, if we could manipulate such relative phases arbitrarily, beyond phase-matching, then we would be able to freely control nonlinear optical processes. Is such control possible in reality? In this Report, we discuss this attractive possibility by using as an example a specific nonlinear optical process, namely a Raman-resonant four-wave-mixing process, including its high-order processes^12^,^13^,^14^.

Before proceeding with the main discussion, we briefly review previous studies related to our work here. The optical-frequency-conversion processes by engineering the quasi phasematching (QPM) gratings to implement sophisticated functions in these processes, have been discussed before. On the basis of such technology, Mizuuchi *et al.* demonstrated the generation of an efficient second harmonic with a broadband spectrum^15^. Recently, Rangelov *et al.* extended this idea to more engineered nonlinear optical-frequency conversions^16^ by applying the concept of composite pulses^17^,^18^. Fejer *et al.* demonstrated the difference-frequency generation accompanying shaped pulse structures^19^. Furthermore, in addition to optical-frequency conversions, nonlinear beam shaping in which two-dimensional computer-generated binary holograms are adopted has been discussed. Shapira *et al.* demonstrated nonlinear mode conversions from a Gaussian beam to Hermite-Gaussian or Laguerre-Gaussian beams^20^.

## Theory

**Figure 1:**
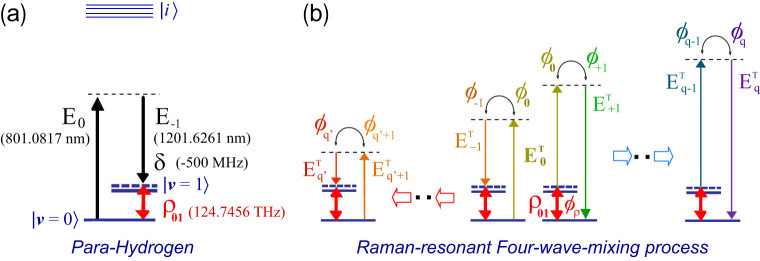
Scheme of Raman-resonant four-wave-mixing process in parahydrogen. (*a*), Adiabatic driving of vibrational coherence at a Raman transition of *v* = 0 to 1. (*b*), High-order four-wave-mixing process initiated from the incident third-laser field, *E_0_^T^*.

Figure 1 illustrates the scheme of the Raman-resonant four-wave-mixing process. We employ gaseous parahydrogen as a nonlinear optical medium and focus on the pure vibrational Raman transition of *v* = 0, *J* = 0 to *v* = 1, *J* = 0 at 125.7451 THz^14^,^21^. First, we adiabatically drive a high coherence between these two vibrational levels, which is achieved by applying two laser-fields, *E_0_* and *E_-1_*, and controlling the small two-photon detuning, *δ*, from the Raman resonance^12^ (Fig. 1). This adiabatic excitation process of high coherence, *ρ_01_*, in turn deeply modulates the two driving laser fields, *E_0_* and *E_-1_* and generates the high-order Stokes and anti-Stokes components, *E_q_* (*q*: integer). The remarkable feature of this nonlinear optical process is that all the high-order components are generated collinearly without being restricted by the (angle) phase-matching condition, because the high coherence produced allows us to efficiently generate the high-order Raman components, *E_q_*, within a unit phase-slip length^12^,^13^,^14^,^22^. Here, we further introduce another laser field, *E_0_^T^*, collinearly with the two driving laser fields, *E_0_* and *E_-1_*. This third laser field is also deeply modulated by the same vibrational motion with high coherence, *ρ_01_* (produced above); moreover, it efficiently generates another series of high-order Stokes and anti-Stokes components, *E_q_^T^* (*q*: integer), also collinearly without being restricted by the phase-matching condition^14^,^23^,^24^.

This nonlinear optical process can be described by using the Maxwell-Bloch equations^12^. A set of density matrix equations, Eq. (1), expresses the optical Bloch equation for coherent vibrational motion in parahydrogen. In this nonlinear optical process, the medium constitutes a so-called far-off resonant *Λ*-scheme^12^,^21^ and can be effectively reduced to a two-level system by defining the two-photon Rabi frequency *Ω_01_*^12^. We assume that the driving and generated Raman fields propagate in the *z* direction, and we use the local-time coordinates *τ* = *t* – *z/c* and *ξ* = *z*. The constant *c* is the velocity of light in a vacuum.
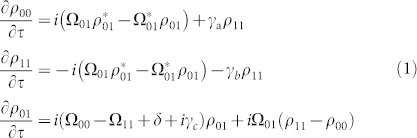
Here, *Ω_00_* and *Ω_11_* are ac-Stark shifts for the states |*v* = 0> and |*v* = 1>, respectively, and *ρ_00_*, *ρ_11_*, and *ρ_01_* are the population of the ground state |*v* = 0>, that of the vibrationally excited state |*v* = 1>, and the coherence associated with this Raman transition, respectively. The coefficients *γ_a_*, *γ_b_* and *γ_c_* are the decay rates of the populations and of coherence, respectively.

The high-order Raman components, *E_q_*, including the two driving-laser fields, *E_0_* and *E_-1_*, propagate in the nonlinear optical medium according to the Maxwell's equation, where all the Raman components are coupled with each other through the Raman coherence, *ρ_01_*, as below:
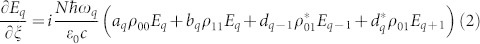
These coupled propagation-equations are expressed in the local-time coordinates with the slowly-varying-envelope-approximation. *E_q_* and *ω_q_* are the electric field amplitude and angular frequency of the *q*th Raman mode, respectively. *N*, *h*, and *ε_0_* are the molecular density, Planck constant, and electric permittivity, respectively. The constants a_q_ and b_q_ determine the dispersions of the medium and d_q_ determines the coupling between neighboring Raman components. (See Refs. 12 and 25 for detailed definitions.) The third laser field, *E_0_^T^*, and its high-order Raman components, *E_q_^T^*, are also in accordance with the same coupled propagation equation as Eq. (2). Here, as described already, the Raman coherence *ρ_01_* is common to the two sets of coupled propagation-equations. However, if we set the third-field amplitude sufficiently weakly compared with those of the two driving fields, then we can treat the behaviors of two series of high-order Raman components, *E_q_* and *E_q_^T^*, almost independently.

In the above framework, we study the artificial manipulation of high-order Stokes and anti-Stokes generation originating in the third laser field, *E_0_^T^*, by controlling the relative phase relationship. To see this phase relationship more explicitly, we transform Eq. (2) to Eq. (3) (*see Supplement for details*):
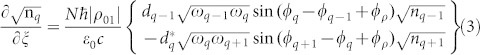
Here, the electric field at the *q*th order, *E_q_*, and the Raman coherence, *ρ_01_*, are expressed explicitly with the amplitude and phase, as *E_q_* = |*E_q_*|exp(*iϕ_q_*), *ρ*_01_ = |*ρ*_01_|exp(*iϕ_p_*). Furthermore, we have changed the expression from one regarding field amplitude, *E_q_*, to photon number-density per mode, *n_q_*. This is done because we want to see this nonlinear optical process from the perspective of “photon flow”. Because the total number of photons is conservative in this nonlinear optical process, the set of coupled propagation-equations in Eq. (3) represents a redistribution motion of photon number-densities among all the generated Raman components, including the incident third laser field, *E_0_^T^*.

As seen clearly in this equation, the directions of these photon flows (the first and second terms are the photon flows to the *q*th order from the (*q* − 1)th and (*q* + 1)th orders, respectively), are determined only by the signs of the relative phases, *ϕ*(*q*, *q* − 1) = *ϕ_q_* − *ϕ_q_*_ − 1_ + *ϕ_ρ_*. Therefore, if we can manipulate such signs, then we can expect to control the “photon flows”. As an example, we study the case in which the target of the photon-flow manipulation is set to the photon-number concentration for a specific Raman mode. The following relative phase or sign relationship is typical for realizing such an aim: mod[*ϕ*(*q* − 1, *q* − 2), 2*π*] = *π*/2, mod[*ϕ*(*q*, *q* − 1), 2*π*] = *π*/2, mod[*ϕ*(*q* + 1, *q*), 2*π*] = −*π*/2 mod[*ϕ*(*q* + 2, *q* + 1), 2*π*] = −*π*/2. This is because, according to Eq. (3), this relative-phase relationship should give a steep photon flow such that (q − 2)th → (q − 1)th → qth ← (q + 1)th ← (q + 2)th. We can naturally expect that all the photons distributed among the high-order Raman modes, (q − 2)th to (q + 2)th, are concentrated to the qth order. If we repeat this relative-phase control by changing the target Raman mode sequentially, we can manipulate the photon flow as intended and thus finally transfer a substantial quantity of the incident photons to the Raman mode of interest.

## Results

### Numerical calculations by assuming arbitrary relative-phase manipulations

To verify this expectation quantitatively, we performed numerical calculations on this nonlinear optical process. Here, we assumed close-to-ideal boundary conditions to confirm the intrinsic potential of the idea as a first step. Namely, we assumed a uniform high Raman coherence of *ρ_01_* = 0.3 in both time and space. We also assumed control of the relative-phases to arbitrary values, which were embedded at the optimal interaction lengths in the respective nonlinear optical processes. Under these assumptions, we numerically solved the coupled propagation-equations for the Raman-resonant four-wave mixing process originating in the third laser field, *E_0_^T^*.

**Figure 2:**
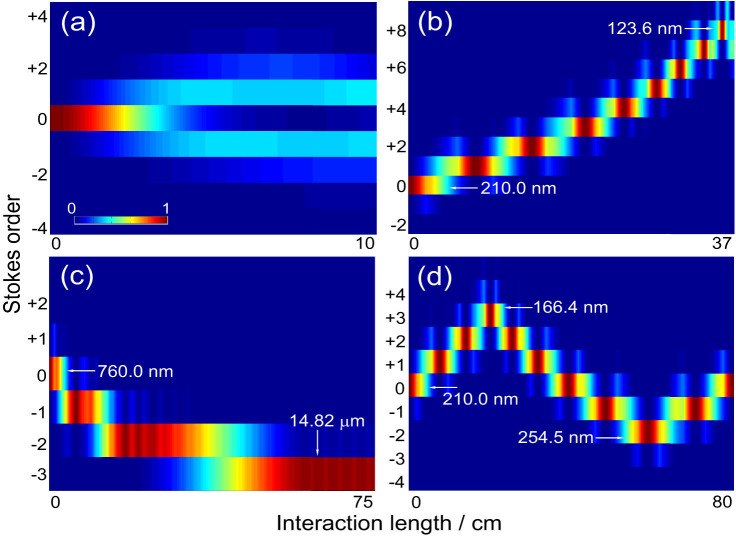
Arbitrary manipulation of Raman-resonant four-wave-mixing processes in parahydrogen. The relative phases among the Raman components, *E_q_^T^*, are assumed to be controlled arbitrarily.

The results in Fig. 2 are typical of those obtained. The molecular density, *N*, and the wavelength of the incident third-laser field were set to 2.6 × 10^18^ *cm^−3^* and 210.0000 nm (Figs. 2a, b, d) or 2.6 × 10^19^ *cm^−3^* and 760.0000 nm (Fig. 2c), respectively. In Fig. 2a, as a reference, no phase manipulation was applied. As already described, the incident photons were distributed broadly to the high-order Raman components. In contrast, in Figs. 2b to 2d various artificial relative-phase manipulations were tested. Such manipulations were performed essentially as indicated above, but, depending on the case we divided the manipulation into more than two steps and thus achieved the desired photon-flows. (*See Supplement for details*). In Fig. 2b the targets were set as the respective high-order Raman modes on the short-wavelength side. As seen clearly, the incident photons were concentrated sequentially down to the 8th order (123.5980 nm), with quantum efficiencies close to unity (>99%). Here, the relative-phase controls were employed discretely 16 times over the whole interaction length. As seen by the clear color-changes, the photons were steeply transferred to the next order around such phase controls. Similarly, we examined the sequential concentrations of photons transferred to the opposite, long-wavelength, side (Fig. 2c). The efficiencies were also close to unity (>98%) for all the steps. In this numerical calculation, the longest wavelength was designed to be 14.8203 μm in mid-infrared. The photon-flow controls are not necessarily restricted to simple unidirectional manipulations like those in Figs. 2b and 2c. Potentially, arbitrary manipulation is possible. The photon-flow manipulation like a wave in Fig. 2d is an example to demonstrate such ability.

### Numerical simulations assuming practically applicable experimental-parameters

Thus, as hoped, we can manipulate photon flows in nonlinear optical processes if we can arbitrarily control the relative phases among the relevant electromagnetic fields. However, how can we achieve such arbitrary phase (or sign) manipulations in reality? Here, we show that they can be realized by using a surprisingly simple technology.

The relative phases among the high-order Raman components can be almost arbitrarily manipulated simply by inserting transparent plates on the optical path and then precisely adjusting their thicknesses (arbitrary optical-phase manipulation by precise control of thickness of a dispersive plate: APM-DiP). This technology can act practically when the frequencies of the relevant electromagnetic fields are discrete, with very large frequency spacings (> tens of terahertz), although it may seem incompatible with the natural physical order. For details, see Refs. 26 to 28; here, we comment only briefly on the key mechanism of the technology. When the frequencies of the relevant electromagnetic fields are very discrete, the thin plates include substantial high-order refractive-index dispersions, and a slight change in their thickness comprehensively sweeps the relative weights among the high-order dispersions. In other words, we can realize almost arbitrary relative-phase relationships.

The critical issue in the actual experiment is that inserting such plates inevitably also affects the process of adiabatic driving of the high coherence, *ρ_01_*, by the two driving-laser fields, *E_0_* and *E_-1_*, where the broad generation of high-order Raman components, *E_q_*, is included self-consistently. However, the coherence itself can be substantially driven even by the two driving-laser fields alone, without accompanying such high-order Raman generation. Thereby, if we drive the coherence in such regime, the relevant phase in this process can be reduced to only one relative-phase between *E_0_* and *E_-1_*, which determines the phase of the coherence, ϕ_ρ_. This implies that the coherence-driving process is essentially not affected by insertion of the dispersive-plates. (*See Supplement for details*). Although ϕ_ρ_ changes steeply around the plates by inserting them and the change of ϕ_ρ_, in turn, affects the photon-flow manipulation among *E_q_^T^*, such influence can be effectively included in the control of the relative-phases, *ϕ*(*q*, *q* − 1) = *ϕ_q_* − *ϕ_q_*_ − 1_ + *ϕ_ρ_*, among *E_q_^T^* for the photon-flow manipulation.

**Figure 3:**
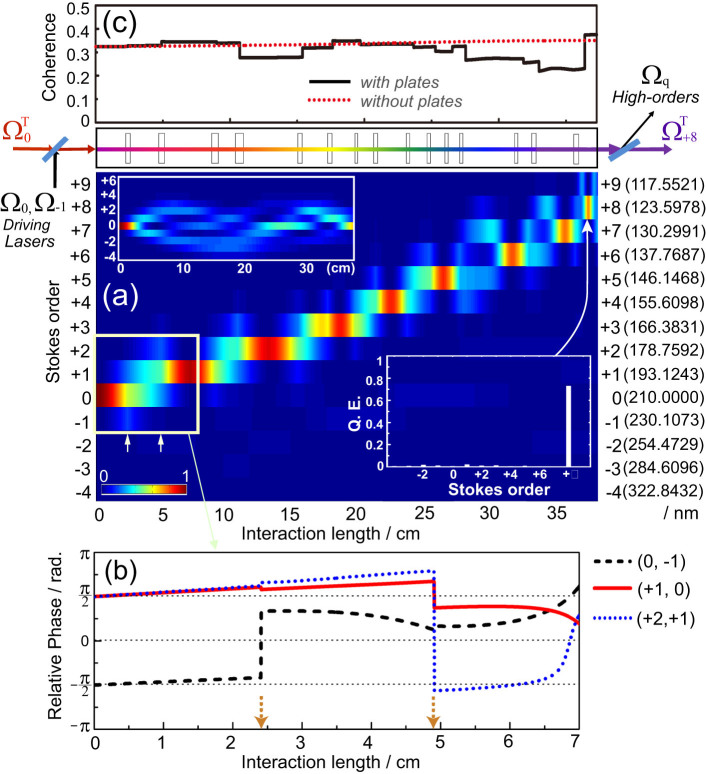
Numerical simulation on artificial manipulation of Raman-resonant four-wave-mixing processes in parahydrogen. (*a*), Contour plot of photon-number distributions among high-order Raman modes. (*b*), Typical example of relative-phase manipulation by inserting magnesium fluoride plates. (*c*), Spatial distribution of vibrational coherence with and without the plates.

Our demonstration is also based on a numerical simulation, the numerical code of which has been verified to be highly reliable^12^,^13^,^14^,^25^. Unlike in the former case (Fig. 2), here we treated all the processes realistically by assuming that the experiment was real. The density of gaseous parahydrogen was set to 2.6 × 10^18^ cm^−3^ and the interaction length to 37.2(95) cm. The vibrational coherence, *ρ_01_* (124.7451 THz), was adiabatically driven from the ground state by the two-color laser fields (*E_0_*: 801.0817 nm; *E_-1_*: 1201.6261 nm; *δ* = −500 MHz). The peak intensity of the driving lasers was set at 10 GW/cm^2^ with a 10-ns pulse duration. The peak intensity of the third laser (*E_0_^T^*: 210.0000 nm; 5 ns) was set at 0.1 GW/cm^2^, 100 times weaker than those of the coherence-driving lasers. The driving and third laser beams were coupled and decoupled in space by setting their polarizations orthogonally (see Fig. 3). As a transparent dispersive material we used magnesium fluoride (MgF_2_) plates (ordinary axis), because they have high transparency in the vacuum-ultraviolet spectral region. For the refractive index dispersion of MgF_2_ we relied on that given by the Sellmeier equation^29^.

To reproduce the optical-frequency conversion demonstrated in Fig. 2b, but under actual experimental conditions, we explored the optimum MgF_2_ plate thicknesses by using the random search method, such that they satisfied the requirements for the relative-phase relationship among *E_q_^T^*. We inserted these plates with the optimum thicknesses at appropriate interaction lengths in gaseous parahydrogen. With this experimental setup, we numerically solved the Maxwell-Bloch equations (Eqs. (1) and (2)) for the Raman-resonant four-wave-mixing processes originating in both the driving lasers and the third laser, including their coupling through the coherence, *ρ_01_*, although the latter is not essential. We did not introduce any artificial assumptions. All of the Raman components were simply retarded in phase to a degree depending on the optical thicknesses of the inserted MgF_2_ plates when they were transmitted the plates.

Figure 3 gives a typical result. Photon flow very similar to that observed in Fig. 2b was reproduced in this numerical simulation. The quantum efficiencies from the incident third laser field to the high-order Raman components were extremely high: 94% at the 1st (193.1243 nm), 84% at the 4th (155.6098 nm) and 73% at the 8th (123.5978 nm). The inset at bottom right of Fig. 3a shows the spectrum generated at an interaction length of 37.2(95) cm. A virtually single line is seen at 123.5978 nm (8th order). To realize this sequential photon-flow manipulation we inserted 15 MgF_2_ plates. The approximate positions of the plates are illustrated in the panel above Fig. 3a. (*See Supplement for details.*) Each plate typically had a position allowance of about ±5 mm and a thickness allowance of about ±0.1 μm, indicating that the technology was practical. Although 15% of incident photons were lost by absorption onto these 15 MgF_2_ plates, this was not a serious problem. This technology is also robust for the physical parameters such as variations in the pump intensity and the molecular density, where an allowance of 5% is enough to keep the conversion efficiency above 90% of the optimum. Figure 3b indicates in detail how we artificially manipulated the relative-phase relationship to realize this photon flow. Only the start of the process is shown (marked with the white square). When the Raman components were transmitted the first MgF_2_ plate, the relative phase between −1st and 0th was changed such that 0 < mod[*ϕ*(0, −1), 2*π*] < *π*. Photon flow to the −1st then flowed back to the 0th as −1 → 0 → +1 → +2. Next, when the Raman components were transmitted the second plate, the relative phase between the +1st and +2nd was controlled as −*π* < mod[*ϕ*(+2, +1), 2*π*] < 0, whereas the others maintained the same sign. The photon flow then changed such that −1 → 0 → +1 ← +2 and finally concentrated at the +1st (193.1243 nm), with 94% concentration at an interaction length of 7.0(8) cm. The inset at top left of Fig. 3a shows the Raman generation with no plates inserted. As in Fig. 2a, the incident photons were naturally distributed broadly to the high-order Raman components. As already described, the above-described phase manipulation by the MgF_2_ plates must not disturb the coherence-driving process by the two driving-laser fields, *E_0_* and *E_-1_*. Figure 3c shows the coherence with and without the plates as a function of interaction length at the peak of the driving pulsed laser-fields. The 15 inserted MgF_2_ plates did not spoil a uniform coherence distribution in space, as expected.

### Applications

**Figure 4:**
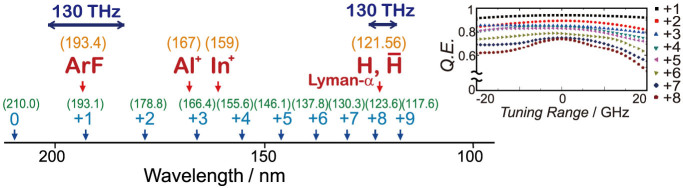
Tunability of high-order Raman lasers produced with the artificial phase-manipulation technology, and possible applications of these single-frequency tunable lasers in the vacuum-ultraviolet region of 120 to 200 nm.

Finally, we show some attractive applications of this technology, including precision spectroscopy in the vacuum-ultraviolet region. The third laser field (210.0000 nm) can be practically generated by taking the fourth harmonic of 840.0000 nm which is produced by an injection-seeded Ti:sapphire laser with a frequency precision of a few MHz^30^. Because the tuning range of this laser can be as wide as ±40 nm^30^, the 210-nm third laser is tunable over ±10 nm, corresponding to a frequency tuning range of 130 THz—greater than the frequency spacing of the present Raman modes (125 THz). Thereby, we can access any wavelengths from 200 to 120 nm in the vacuum-ultraviolet region. New-wavelength selection requires additional exploration of the optimum thicknesses of the inserted MgF_2_ plates. However, if the tuning range is within ±20 GHz (sufficient for various spectroscopic applications) we need not adjust the plate thicknesses (see inset in Fig. 4 which shows quantum efficiencies of the high-order Raman generations where the plate thicknesses were fixed). We also note that, besides this arbitrary wavelength selectivity, this laser technology has other attractive abilities, such as high spectral intensity enabling nonlinear spectroscopy, high frequency precision derived from an optical-frequency-standard precision^31^,^32^, and scalability to ultrahigh energy (e.g. >1 Joule per pulse).

Some attractive candidates for application are shown in Fig. 4. They include single-ion spectroscopy for optical-frequency standards: ^*1*^*S_0_* to ^*1*^*P_1_* transition of Al^+^ (167 nm)^33^ and that of In^+^ (159 nm)^34^; laser cooling of hydrogen and antihydrogen for testing the standard theory: Lyman-α transition at 121.56 nm^35^,^36^. Scalability to ultrahigh power will also be attractive from an industry perspective (e.g. high-average-power 193-nm laser for lithography).

## Discussion

In summary, we have described the physics of arbitrary manipulations of nonlinear optical processes. By employing a Raman resonant four-wave mixing process in gaseous parahydrogen, we showed that this nonlinear optical process can be freely controlled by manipulating the relative-phase relationship among the relevant electromagnetic fields. Furthermore, we showed that these arbitrary manipulations can be realized by using an extremely simple technology that inserts transparent dispersive materials (MgF_2_ plates) in the nonlinear optical medium and adjusts their positions and thicknesses precisely. As a typical example, we demonstrated freely designable optical-frequency conversions with near-unity quantum efficiencies of 73% to 94%; they covered the whole vacuum-ultraviolet spectral range of 120 to 200 nm with a continuous frequency-tunability of more than ±20 GHz.

The concept described in this Report can be applied to various nonlinear optical processes with various configurations, such as intracavity geometry. Harmonic generations including high-order harmonics, and soliton generation will be attractive candidates. We are currently performing an experiment to verify this concept.

## Author Contributions

M.K. directed the project and proposed the conceptual idea on “freely designable optical frequency conversion process”. J.Z. systematically performed the computations. M.K. and J.Z. intensively discussed various aspects on the computational results and co-wrote the manuscript.

## Supplementary Material

Supplementary Information Supplementary Information
