# Supplementary material for: Freely designable optical frequency conversion in Raman-resonant four-wave-mixing process
Source: Sci Rep. 2015 Mar 9;5:8874. doi: 10.1038/srep08874 (PMC4352894; doi:10.1038/srep08874)
Supplement: Supplementary Information [file srep08874-s1.pdf]

Supplementary Information for the paper:

Freely designable optical frequency conversion in Raman-resonant four-wave-mixing process

Jian Zheng[1] **& Masayuki Katsuragawa**[1, 2, *]

[1]Department of Engineering Science, University of Electro-Communications

[2]JST ERATO-IOS

1-5-1, Chofugaoka, Chofu, Tokyo 182-8585, Japan

*katsuragawa@uec.ac.jp

<u>Derivation of the expression of the coupled propagation-equation in terms of ‘photon number-density per mode’</u>

Substitute

$$E_q = \left|E_q\right| \exp\left(i f_q\right), \quad r_{01} = \left|r_{01}\right| \exp\left(i f_r\right)$$

into the coupled propagation-equation for the Raman mode q.

$$\frac{\partial E_q}{\partial x} = i \frac{N\mathbf{h} w_q}{e_0 c}\left(a_q r_{00} E_q + b_q r_{11} E_q + d_{q-1} r_{01}^{*} E_{q-1} + d_q^{*} r_{01} E_{q+1}\right)$$

We obtain

$$\frac{\partial E_{\mathrm{q}}}{\partial x} + i\left|E_q\right| \frac{\partial f_{\mathrm{q}}}{\partial x} = \mathrm{i}\, \frac{\mathrm{N}\mathbf{h} w_q}{e_0 \mathrm{c}}\left(a_q r_{00}\left|E_q\right| + b_q r_{11}\left|E_q\right| + d_{q-1}\left|r_{01}\right|\left|E_{q-1}\right| \exp\left(i f_{q-1} - i f_q - i f_r\right) + d_{q}^{*}\left|r_{01}\right|\left|E_{q+1}\right| \exp\left(i f_{q+1} - i f_q + i f_r\right)\right)$$

Then, we arrange this equation separately for each of the real and imaginary parts. The following two equations are reduced.

$$\frac{\partial E_{\mathrm{q}}}{\partial x} = \frac{\mathrm{N}\mathbf{h} w_q}{e_0 \mathrm{c}}\left(d_{q-1}\left|r_{01}\right|\left|E_{q-1}\right| \sin\left(f_q - f_{q-1} + f_r\right) + d_{q}^{*}\left|r_{01}\right|\left|E_{q+1}\right| \sin\left(f_{q+1} - f_q + f_r\right)\right)$$

$$\frac{\partial f_{\mathrm{q}}}{\partial x} = \frac{\mathrm{N}\mathbf{h} w_q}{e_0 \mathrm{c}}\left(a_q r_{00} + b_q r_{11} + d_{q-1} \frac{\left|E_{q-1}\right|}{\left|E_q\right|}\left|r_{01}\right| \cos\left(f_q - f_{q-1} + f_r\right) + d_q^{*} \frac{\left|E_{q+1}\right|}{\left|E_q\right|}\left|r_{01}\right| \cos\left(f_{q+1} - f_q + f_r\right)\right)$$

Furthermore, we change the expressions from one regarding electric field amplitude, E_q, to photon number-density per mode, n_q.

Here, $\sqrt{n_q} \propto \frac{\left|E_q\right|}{\sqrt{\mathbf{h} w_q}}$. Thus, we obtain

$$\frac{\partial\sqrt{n_{\mathrm{q}}}}{\partial x}=\frac{\mathrm{N}\mathbf{h}\left|r_{01}\right|}{e_0\mathrm{c}}\left(d_{q-1}\sqrt{w_{q-1}w_q}\sin\left(f_q-f_{q-1}+f_r\right)\sqrt{n_{q-1}}-d_q^{*}\sqrt{w_qw_{q+1}}\sin\left(f_{q+1}-f_q+f_r\right)\sqrt{n_{q+1}}\right)$$

$$\frac{\partial f_{\mathrm{q}}}{\partial x}=\frac{\mathrm{N}\mathbf{h}\left|r_{01}\right|}{e_0\mathrm{c}}\left(a_qw_q\frac{r_{00}}{\left|r_{01}\right|}+b_qw_q\frac{r_{11}}{\left|r_{01}\right|}+d_{q-1}\sqrt{w_{q-1}w_q}\cos\left(f_q-f_{q-1}+f_r\right)\sqrt{\frac{n_{q-1}}{n_q}}+d_q^{*}\sqrt{w_qw_{q+1}}\cos\left(f_{q+1}-f_q+f_r\right)\sqrt{\frac{n_{q+1}}{n_q}}\right)$$

<u>Details of the relative-phases employed in Figs. 2*b*-*d*.</u>

There are various ways of realizing the same results by using methods other than this relative-phase relationships. However, for practical systems, the simpler the control, the better. In this respect, the step-by-step photon-transfer approach described here would be one of the best, because it requires a minimum number of relative-phase controls (typically two). The red color indicates the relative phases for the Raman modes to be substantially controlled in order to realize the photon number-density concentrations.

Table 1: Relative phases employed in Fig. 2b.

Step	Position (cm)	Relative Phase (radian)												
		f (-3,-4)	*f* (-2,-3)	*f* (-1,-2)	*f* (0,-1)	*f* (1,0)	*f* (2,1)	*f* (3,2)	*f* (4,3)	*f* (5,4)	*f* (6,5)	*f* (7,6)	*f* (8,7)	*f* (9,8)
0 ⇩	1.975	2.92	-1.41	0.68	0.876	0.775	-3.14	2.11	1.89	-2.65	1.13	2.45	-1.79	-0.796
1	5.565	1.23	-0.328	0.881	1.19	1.13	-2.07	-2.14	-2.17	-1.57	-2.91	2.25	1.97	1.14
1 ⇩	9.6	3.1	-0.224	2.17	0.807	0.824	0.993	-2.44	2.15	1.42	-0.179	-0.738	0.158	-3.05
2	12.485	-2.98	-2.01	0.351	-0.731	1.03	1.2	-2.01	-2.09	-2.08	-2.4	-2.17	1.3	-2.94
2 ⇩	15.765	1.68	-0.713	-1.58	2.76	0.67	0.421	0.303	-3.12	2.83	-0.793	-2.74	-0.25	-3
3	18.465	0.681	-1.3	-2.17	0.616	1.43	1.12	1.13	-2.11	-2.22	-2.33	-2.25	-3.05	-2.48
3 ⇩	21.255	-1.19	-1.55	-1.46	1.82	-2.83	0.836	0.795	1.02	2.76	1.8	-0.00439	1.91	2.04
4	23.245	0.81	2.74	-2.64	0.642	-2.91	1.42	1.08	1.16	-2.09	-2.19	-2.22	-2.19	1.62
4 ⇩	25.315	0.821	-1.24	-2.11	-2.51	0.98	1.35	-1.37	0.979	0.97	0.826	2.66	-0.221	-1.38
5	26.905	2.31	0.444	-2.74	-1.57	-2.75	-2.97	1.18	1.09	1.06	-2.26	-2.39	-2.69	2.94
5 ⇩	28.89	-1.23	-0.712	0.405	1.59	-2.66	1.63	-2.96	0.972	0.883	0.823	-2.29	2.18	1.83
6	30.18	-2.24	2.92	1.16	-1.9	-1.89	-2.6	-2.63	0.223	1.22	1.19	-2.11	-2.24	-2.77
6 ⇩	31.715	0.181	0.528	1.8	0.0656	0.5	-2.03	-2.75	-1.57	1.06	1.04	1.18	2	3.11
7	33.215	1.02	-2.73	2.92	0.223	-1.28	1.01	-1.78	0.802	2.65	1.43	1.28	-2.01	-2.23
7 ⇩	34.23	-0.502	1.22	2.28	1.09	-0.236	0.304	3.1	-2.86	0.849	0.437	0.227	-0.46	-1.85
8	35.595	-0.526	1.77	-0.189	0.762	-1.44	-3.08	-2.3	-2.05	0.809	-0.0494	-1.62	1.52	-1.72

Table 2: Relative phases employed in Fig. 2c.

Step	Position (cm)	Relative Phase (radian)					
		f (-2,-3)	*f* (-1,-2)	*f* (0,-1)	*f* (1,0)	*f* (2,1)	*f* (3,2)
0 ↓ -1	12.38	-0.668	-2.74	-2.88	-2.31	-2.62	-0.403
	23.82	-1.11	-1.91	-2.6	-3.02	2.55	2.82
	35.125	-0.168	-0.725	-2.8	-3.08	2.52	1.76
	47.81	-0.369	0.238	-2.97	-2.9	-2.25	1.73
-1 ↓ -2	78.275	-0.908	3.14	2.72	2.22	0.222	0.893
	94.68	1.02	-3.13	2.97	1.27	-2.89	2.68
	110.74	0.628	3.12	-2.72	-2.91	2.43	-0.309
	126.845	2.88	3.11	3.12	2.81	1.72	2.64
	143.28	-0.976	3.13	3.1	-2.91	-0.583	-1.03
	159.45	0.0805	-3.12	-3.1	-2.33	-2.18	0.718
-2 ↓	190.445	3.07	-2.92	2.8	-2.13	-2.92	2.79
	207.81	3.03	1.9	1.76	-1.66	-3.11	-2.84
	224.895	3.13	-2.91	-2.62	-1.48	-2.41	-3.13
	241.315	3.11	2.25	0.762	1.39	2.34	-2.8
	258.11	3.07	-2.97	-2.01	-0.19	2.62	-0.399
	274.775	-3.11	2.99	0.944	2.31	-3.13	-0.888
	290.91	3.11	-2.74	1.66	-2.2	2.98	0.715
	307.275	-3.12	2.67	2.57	1.75	-2.35	0.576
	323.43	3.09	-2.76	2.74	-2.32	2.75	1.73
	340.445	3.08	-2.55	-3.06	1.76	-0.857	-0.118
	356.92	-3.06	-2.42	3.11	-0.786	-2.37	2.57
	372.455	3.1	-2.82	2.81	-2.45	1.88	-1.84
	389.09	3.07	-2.84	-3.1	1.59	-1.57	2.45

	406.23	3.07	-2.53	-3.14	-0.652	-3.01	-1.25
	422.855	3.08	-2.5	2.84	-2.23	1.81	1.4
	439.6	3.06	-2.62	-3.04	2.26	-2.15	0.086
	457.065	3.03	-2.76	-3	1.19	2.86	2.47
	473.825	3.13	-2.56	2.84	-0.619	1.23	-1.11
	489.865	-3.11	-2.6	2.83	-3.11	-1.84	2.77
	506.16	3.06	-2.72	-3.12	1.42	2.52	-1.56
	522.78	3.09	-2.75	-3.12	-1.09	0.313	2.89
	539.025	3.11	-2.67	-2.33	2.27	-2.2	0.353
	555.59	3.07	-2.62	-2.16	0.231	2.64	2.14
-3	572.485	3.1	-2.82	2.44	0.0399	-0.287	-0.34

As shown here, the concentration of photons transferred to the long-wavelength side required many more relative-phase manipulation steps, because the ratios of the nonlinear coupling-coefficient to the linear dispersion-coefficient decreased as the relevant laser-wavelength increased.

Table 3: Relative phases employed in Fig. 2d.

Step	Position (cm)	Relative Phase (radian)								
		f (-3,-4)	*f* (-2,-3)	*f* (-1,-2)	*f* (0,-1)	*f* (1,0)	*f* (2,1)	*f* (3,2)	*f* (4,3)	*f* (5,4)
0 ⇩	1.975	2.92	-1.41	0.68	0.876	0.775	-3.14	2.11	1.89	-2.65
1	5.565	1.23	-0.328	0.881	1.19	1.13	-2.07	-2.14	-2.17	-1.57
1 ⇩	9.6	3.1	-0.224	2.16	0.807	0.824	0.993	-2.44	2.15	1.42
2	12.485	-2.98	-2.01	0.351	-0.731	1.03	1.2	-2.01	-2.09	-2.08
2 ⇩	15.765	1.68	-0.713	-1.58	2.76	0.67	0.421	0.303	-3.12	2.83
3	18.465	0.681	-1.3	-2.17	0.616	1.43	1.12	1.13	-2.11	-2.22
3 ⇩	21.405	-1.73	2.45	-2.36	3.1	-0.986	1.08	-2.05	-2.29	-2.41
2	23.955	0.769	1.12	-2.92	2.87	1.29	1.22	-1.99	-2.05	-2.67
2 ⇩	27.03	2.52	-1.92	1.27	-2.06	1.66	-2.48	-2.33	-2.47	-2.61
1	30	2.8	-1.19	1.59	1.26	1.21	-1.99	-2.04	-2.11	-2.83
1 ⇩	33.775	2.28	-0.27	-0.721	-0.0402	-2.07	-2.58	-2.63	-2.73	-0.948
0	37.18	2.18	2.17	1.18	1.2	-2.06	-1.95	-2.24	-2.08	0.899
0 ⇩	41.695	2.85	2.14	2.24	-2.56	-2.27	-2.28	-2.44	0.352	1.29
-1	45.815	-1.23	1.16	1.15	-2.03	-2.06	-2.16	-3.05	0.49	0.708
-1 ⇩	51.225	-0.221	1.18	-2.43	-2.6	-2.66	-2.58	-1.9	0.878	-1.46
-2	56.215	3.14	1.19	-2.01	-1.95	-1.87	2.8	-1.92	1.32	1.43
-2 ⇩	61.665	0.796	0.784	1.23	-2.73	-1.08	-0.563	-0.634	0.0952	2.22
-1	67.605	1.34	1.11	1.08	-2.13	-2.17	-2.43	0.637	1.01	-1.75
-1 ⇩	73.09	0.864	0.856	0.814	1.05	-2.88	0.0276	-1.71	-1.93	-2.3
0	77.4	-0.0809	1.63	1.19	1.15	-2.05	-2.09	-2.11	2.25	-0.701

Positions and thicknesses of MgF_2 plates inserted in gaseous parahydrogen in Fig. 3.

Index	Position (cm)	Thickness (mm)
1	2.360	0.52591
2	4.925	0.70358
3	8.960	0.78121
4	10.700	0.92920
5	15.275	0.50902
6	17.525	0.51987
7	19.585	0.20664
8	21.150	0.20680
9	23.585	0.33349
10	25.305	0.33218
11	26.550	0.37618
12	27.570	0.35804
13	31.770	0.37017
14	32.835	0.46953
15	36.305	0.23209

Comment on simultaneous control of the two types of relative-phases: one regarding photon-flow manipulation and the other regarding coherence-driving process

The coherence-driving process constitutes a physically self-consistent system with the high-order Raman components, E_q, together with their relative phases, which are typically as follows: *mod*[f (0, -1), 2π] = -π/2, *mod*[f (+1, 0), 2π] = π/2, *mod*[f (+2, +1), 2π] = π/2. According to Equation 3, this relative-phase relationship gives a photon flow from the initial 0th to the neighboring orders ($-1 \leftarrow 0 \rightarrow 1 \rightarrow 2$), like a "diffusion process", corresponding to a broad Raman generation on both the short- and the long-wavelength side; this differs completely from the "photon-flow manipulation" in Figure 2. (In other words, it is difficult to implement photon-flow manipulations like those in Figures 2*b* t*o 2d* together with adiabatic driving of coherence in a single nonlinear optical process.) Ideally, the insertion of the dispersive plates should satisfy this relative-phase requirement too, simultaneously with those required for photon-flow manipulations (e.g. those in Figs. 2*b* to *2d*). This is also technically possible. One way of achieving this ideal condition is to set the wavelengths and/or polarizations so that they differ between the two series of high-order Raman components, E_q^T and E_q, and then apply the technology: APM-DiP for E_q^T and E_q, respectively.
